# Improving biodiversity assessment via unsupervised separation of biological sounds from long-duration recordings

**DOI:** 10.1038/s41598-017-04790-7

**Published:** 2017-07-03

**Authors:** Tzu-Hao Lin, Shih-Hua Fang, Yu Tsao

**Affiliations:** 1 Research Center for Information Technology Innovation, Academia Sinica, Taipei, Taiwan (R.O.C.); 2 Department of Electrical Engineering, Yuan Ze University, Taoyuan, Taiwan (R.O.C.)

## Abstract

Investigating the dynamics of biodiversity via passive acoustic monitoring is a challenging task, owing to the difficulty of identifying different animal vocalizations. Several indices have been proposed to measure acoustic complexity and to predict biodiversity. Although these indices perform well under low-noise conditions, they may be biased when environmental and anthropogenic noises are involved. In this paper, we propose a periodicity coded non-negative matrix factorization (PC-NMF) for separating different sound sources from a spectrogram of long-term recordings. The PC-NMF first decomposes a spectrogram into two matrices: spectral basis matrix and encoding matrix. Next, on the basis of the periodicity of the encoding information, the spectral bases belonging to the same source are grouped together. Finally, distinct sources are reconstructed on the basis of the cluster of the basis matrix and the corresponding encoding information, and the noise components are then removed to facilitate more accurate monitoring of biological sounds. Our results show that the PC-NMF precisely enhances biological choruses, effectively suppressing environmental and anthropogenic noises in marine and terrestrial recordings without a need for training data. The results may improve behaviour assessment of calling animals and facilitate the investigation of the interactions between different sound sources within an ecosystem.

## Introduction

Evaluating the effects of environmental change and human development on biodiversity is a critical task in conservation management^1, 2^. Assessment of biodiversity requires a significant amount of resources to collect samples and investigate the presence of animals. Owing to limited resources, the temporal dynamics of biodiversity is usually difficult to measure, especially in remote areas where surveys are conducted seasonally or annually. Therefore, time series information of biodiversity is a much more challenging area of study than geographical information^3^.

In recent years, passive acoustics has become a popular method for monitoring the behaviour of wildlife. By using autonomous sound recorders, vocalizations of calling animals can be collected with long durations in various environments, such as deep waters and tropical rain forests^4–6^. Despite achieving the collection of terabytes of data, difficulties remain in identifying all calling animals, owing to the complexity of animal vocal behaviours. Instead of trying to identify all calling species, several ecoacoustics indices have been developed to evaluate the biodiversity changes on the basis of acoustic data. To name just a few, these indices include the acoustic entropy index^7^, acoustic diversity index^8^, acoustic complexity index^9^, and acoustic richness^10^. Although the measurements vary among indices, all of these indices intend to quantify the spectral and temporal complexity of biological sounds.

Ecoacoustics indices have been used to investigate the composition of calling animals, interactions among different sound sources and their relationships within their physical and biological environments^11–13^. Several studies have also successfully tested the correlations between ecoacoustics indices and local biodiversity in tropical forests, temperate forests, and coastal reef environments^7, 10, 14^. However, not all indices perform equally regarding the correlations of biodiversity changes. Some of the indices are sensitive to the environmental noise caused by the unique landscape characteristics and the presence of anthropogenic noise^15^. Therefore, the uncertainty increases when ecoacoustics indices are used to investigate the spatial and temporal changes in biodiversity in noisy environments.

The uncertainty of ecoacoustics indices in noisy environments can be decreased if noise can be effectively suppressed before acoustical analysis. Various noise reduction (NR) methods have been developed in the field of acoustic signal processing and analysis. Among the NR methods, a notable class is the spectral restoration methods. The goal of the spectral restoration methods is to separate the noise-free signals from the given noise-corrupted ones in the spectral domain. Well-known spectral restoration approaches include spectral subtraction (SS)^16^ and the Wiener filter^17^, with their various extensions^18, 19^. Another group of spectral restoration methods is based on the statistical models of noise-free and noise-corrupted signals to derive a gain function, which is used to filter noise-corrupted input signals to obtain the noise-free counterparts. Notable examples include the minimum-mean-square-error (MMSE)^20^, maximum likelihood spectral amplitude estimator (MLSA)^21^, maximum a posteriori spectral amplitude (MAPA)^22^, and generalized maximum a posteriori spectral amplitude (GMAPA)^23^. The spectral restoration methods usually rely on a noise tracking scheme to calculate the statistics of noise components; effective noise tracking schemes include minimum statistics (MS) tracking^24^, minima controlled recursive averaging (MCRA)^25^, and improved MCRA (IMCRA)^26^. Although the conventional spectral restoration methods can provide satisfactory performance under most conditions, there is still room to further improve them for non-stationary noisy conditions. In field recordings, both environmental and anthropogenic noises are usually non-stationary. Therefore, the conventional spectral restoration methods may not be sufficient for improving the performances of ecoacoustics indices.

More recently, the non-negative matrix factorization (NMF) algorithm has been adopted for noise reduction and shows promising performance in handling non-stationary noises^27, 28^. The NMF has been used to model the encoded information of an acoustic signal and noise in a noisy recording. By modelling the signal-related features and encoding matrix, the acoustic signal can be separated or enhanced from the noisy recordings^29^. Although the NMF achieves satisfactory performance in many acoustic noise reduction tasks, a major limitation to its applicability is that a set of supervised training data must be prepared to achieve optimal performance. More specifically, pure signal sources must be collected in the offline phase to facilitate effective noise reduction in the online phase. Under real-world conditions, however, collecting pure signals may not always be possible. Therefore, the traditional supervised NMF method cannot be suitably used in the passive acoustic monitoring of biodiversity.

In this study, we sought to address the limitations of conventional supervised NMF noise reduction methods, and we propose a novel NMF with periodicity analysis (PC-NMF) for suppressing noises from spectrograms of long-duration recordings in an unsupervised manner. On the basis of the periodicity of encoded information, PC-NMF can separate multiple biological choruses and suppress noise signals. In the experiment, we first evaluated the performance of PC-NMF on a set of simulated test data. For comparison, we also conducted experiments using supervised NMF and several well-known spectral restoration methods. Then, the PC-NMF was applied to real-world data collected in terrestrial and marine environments with varying noise levels. Finally, we discuss the potential application of PC-NMF in future research of ecoacoustics.

## Results

### Performance evaluation on the basis of simulation data

**Figure 1:**
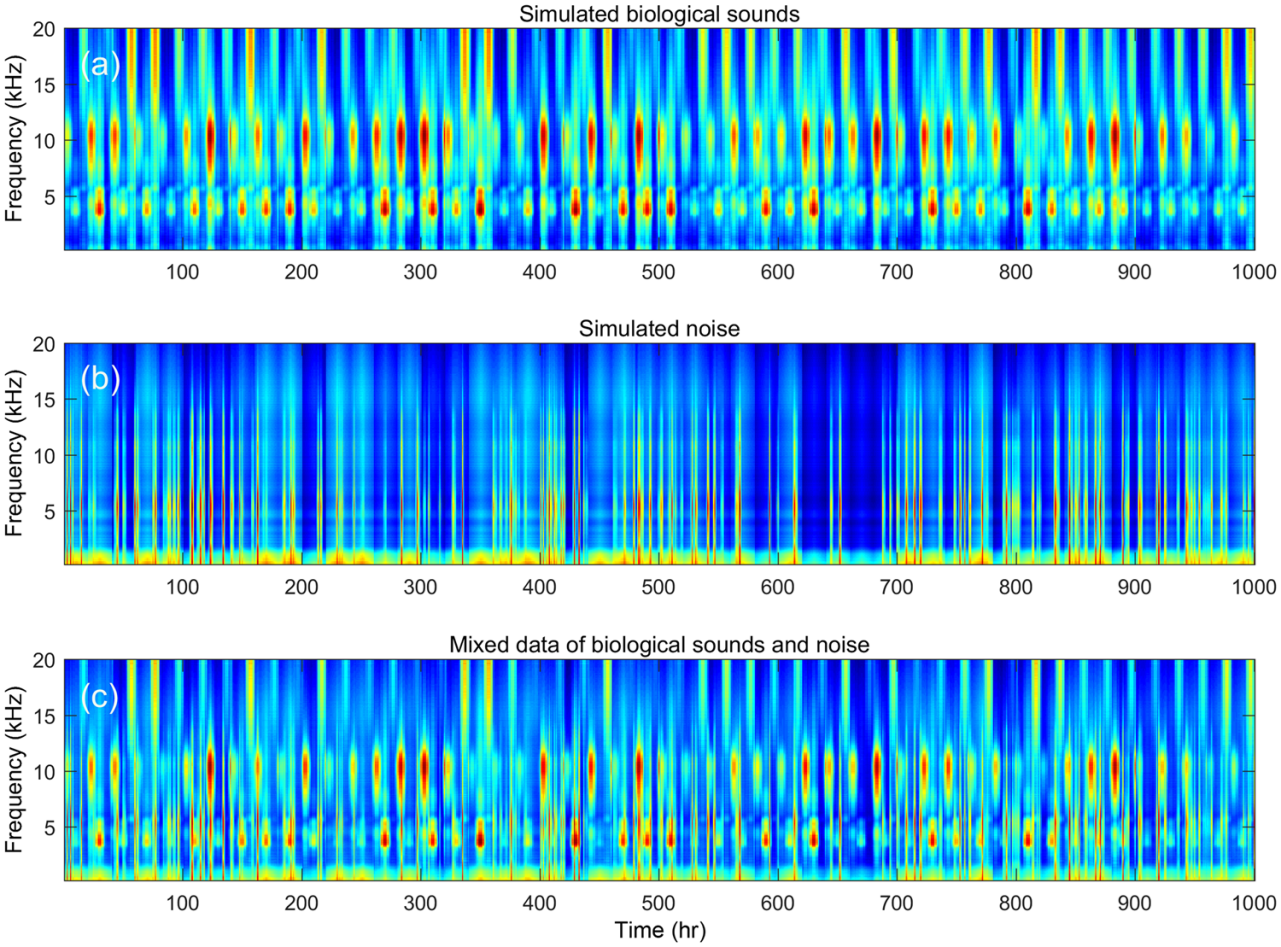
Simulation spectrogram used in this study. (**a**) The combined data of three types of biological sounds; (**b**) the combined data of two different types of noises; (**c**) a mixture of the data from (**a**) and (**b**).

**Figure 2:**
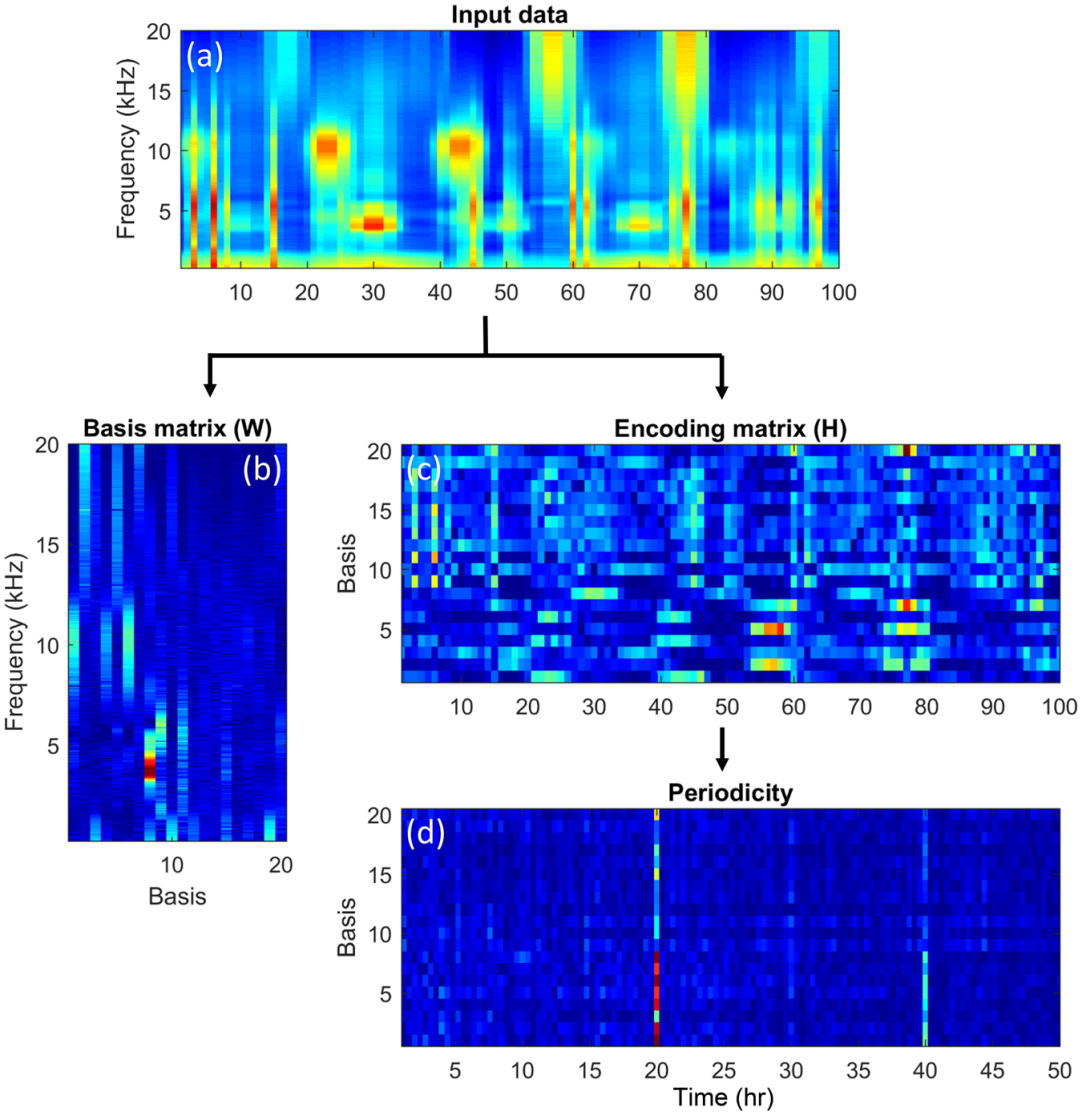
An example of factorizing a spectrogram by using the PC-NMF. (**a**) Part of the simulation data; (**b**) spectral basis matrix; (**c**) encoding matrix. The encoding matrix was transformed (using discrete Fourier transform) to obtain (**d**) the periodicity matrix. The prominent peak and harmonic structures of the first eight bases in the periodicity matrix indicate that they represent the bases of a biological chorus.

To evaluate the performance of PC-NMF, we prepared three types of biological sounds (Fig. 1a), one type of transient broadband noise and one type of environmental noise (Fig. 1b), and a mixture of these five sources to use in our simulated long-term spectrogram (Fig. 1c). During the analysis, we first factorized the spectrogram into a basis matrix and an encoding matrix via NMF (Fig. 2). The basis matrix represented the spectral dictionary of the input spectrogram, and each sound source appeared to be composed of multiple bases. The encoding matrix described the occurring intensity of each basis along the time axis. It was obvious that some bases related to the three biological sounds displayed stronger periodicity than those related to noise sources (Fig. 2d). Therefore, we grouped the bases that shared similar periodicity by analysing the periodicity of the encoding matrix, and each group represented the bases of a distinct sound source. Finally, the biological chorus was reconstructed according to the bases with strong periodicity and the corresponding encoding information, and non-periodical noise was effectively suppressed.

We first quantitatively analysed the performance of PC-NMF on the basis of the spectral similarity metric, an index ranging from −1 to 1, to evaluate the precision of the noise reduction result of different methods. In a preliminary experiment, we tested the performances of PC-NMF with different basis numbers and iteration numbers using the simulated spectrogram. The results showed that the spectral similarity reached 0.78 and became saturated when the basis number was larger than 60. Notably, when a larger basis number was used, the computational power was accordingly increased. To strike a reasonable balance between performance and computation, we report only the results of using 60 basis numbers in the following results. However, we noted that the spectral similarity increased accordingly when the number of iterations increased from 80 to 200, whereas the performance of the spectral similarity became saturated after 200 iterations. In the following discussions, we report the results for 200 iterations.

**Figure 3:**
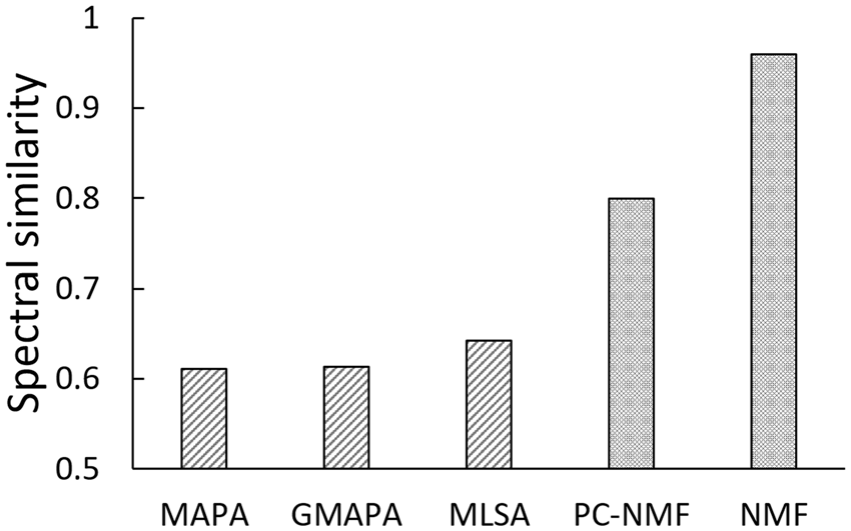
The noise reduction performances of different methods. A higher spectral similarity suggests better noise reduction performance. The bars represent the mean value of spectral similarity calculated after 20 repeated measurements.

Next, we compared PC-NMF with the supervised NMF and several conventional spectral restoration methods, including MLSA, MAPA, and GMAPA. The results are presented in Fig. 3. From the results, we observed that the supervised NMF achieved the highest performance in separating biological sounds from the simulated spectrogram: when the training data of biological sounds and noise were given, the spectral similarity reached 0.95. However, the spectral similarity of PC-NMF reached 0.8 (Fig. 3). Although PC-NMF did not perform as well as the supervised NMF, it is still promising, because it provides a noise reduction performance when no supervised training data are given. Finally, the results in Fig. 3 show that the PC-NMF outperformed the conventional MLSA, MAPA, and GMAPA spectral restoration methods (no training data were used in these three methods).

**Figure 4:**
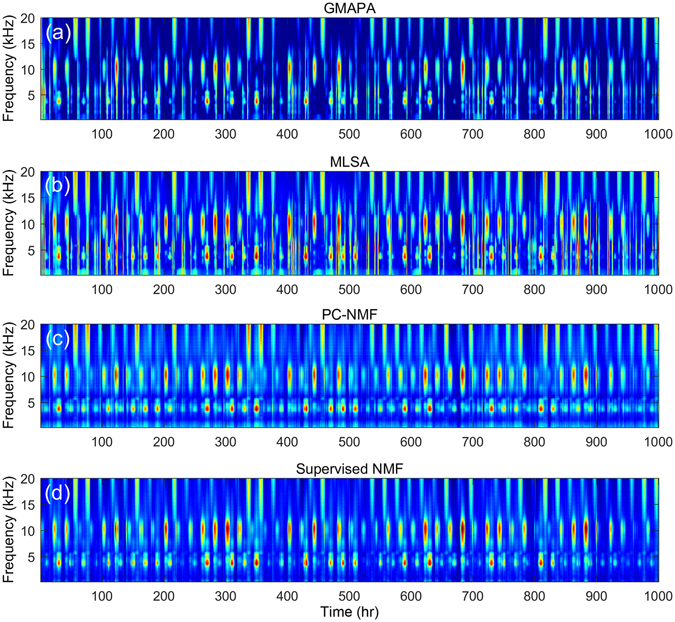
Results of four noise-suppression methods. Spectrograms of noise reduction results generated with (**a**) GMAPA, (**b**) MLSA, (**c**) PC-NMF, and (**d**) supervised NMF.

Next, we sought to qualitatively evaluate the effectiveness of PC-NMF by using the simulated test data. Figure 4 shows the spectrograms processed by using GMAPA, MLSA, PC-NMF, and the supervised NMF in (a)-(d), respectively. From the figure, it is clear that the conventional spectral restoration methods (GMAPA and MLSA) could not effectively discriminate the biological sounds and transient noises. Therefore, some parts of the biological sounds were distorted, and most of the transient noises were even magnified when the conventional NR methods were used. Next, in comparison with the results in Figs 1a and 4c, PC-NMF effectively suppressed the background noise while maintaining clear structures of the biological sounds. Finally, in comparing Fig. 4c and d, we found that the spectrogram generated by PC-NMF (in an unsupervised manner) approached that generated by the supervised NMF (which required pure training data).

### Experimental results based on real-world data

To evaluate the performance of PC-NMF for real-world data, we prepared four datasets collected from marine and terrestrial environments. In the real-world scenarios, we were not able to record pure biological sounds without any noise interference. Thus, it was impossible to evaluate the performance with respect to spectral similarity (which would require pure sources as a reference). Instead, we measured the sound intensity in each time vector of the separated data as a quantitative index for assessing the performance. The sound intensity was normalized by dividing the intensity of each 5-minute recording fragment by the maximum intensity of the entire data.

**Figure 5:**
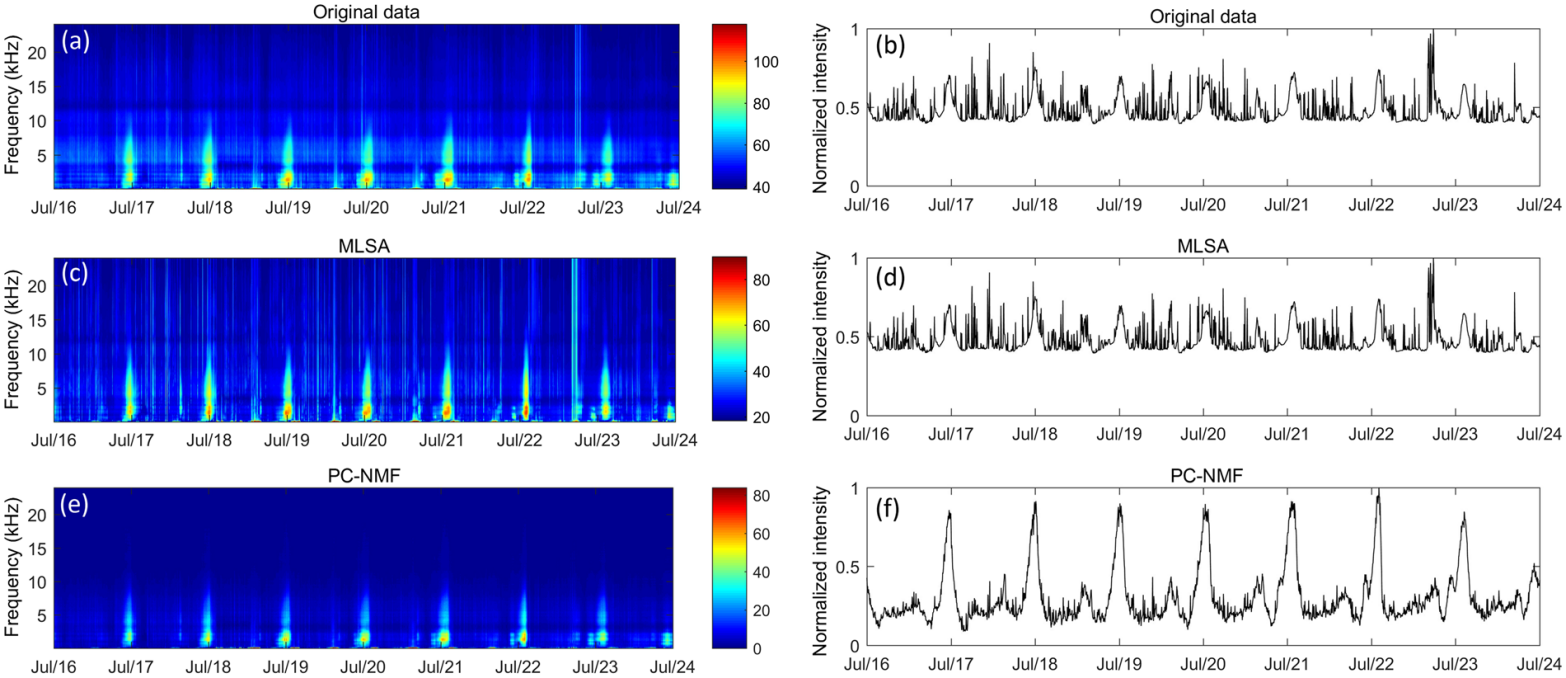
Spectrograms and normalized intensities of underwater recordings collected at the Waisanding Sandbar. The graph shows a comparison between (**a**,**b**) the original data and (**c**,**d**) the analysis results using the MLSA method and (**e**,**f**) the PC-NMF.

**Figure 6:**
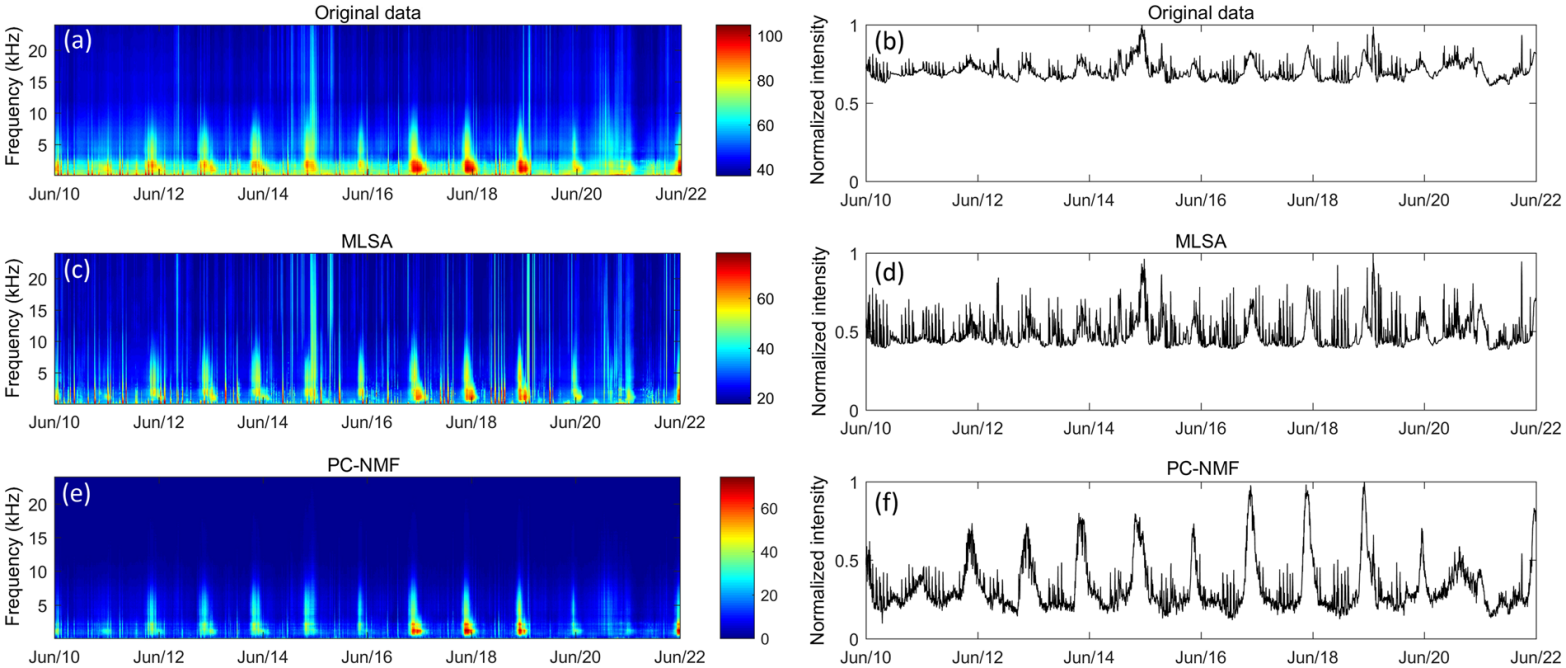
Spectrograms and normalized intensities of underwater recordings collected at the Xinhuwei River Estuary. The graph shows a comparison between (**a**,**b**) the original data and (**c**,**d**) the analysis results using the MLSA method and (**e**,**f**) the PC-NMF.

The results are shown in Figs 5 and 6. As shown in the figures, we observed that the PC-NMF was capable of enhancing biological choruses with strong periodicity under different noise conditions. In shallow marine environments, the primary biological sound is the night-time fish chorus, as shown in both Figs 5 and 6. In both the pristine area and the river estuary near an industrial harbour, the signal-to-noise ratios of fish choruses were relatively low, owing to the presence of stationary environmental noise and transient shipping noise.

The conventional spectral restoration method (only MLSA results are displayed here, although MAPA and GMAPA showed similar trends) successfully removed stationary environmental noise, but it was inefficient in suppressing the transient broadband noise. By using the PC-NMF, we found that the fish choruses were considerably enhanced for both recording locations (Figs 5, 6). In addition to the stationary environmental noise, PC-NMF showed a promising performance in suppressing the transient broadband noise. Similar performances were also achieved for the other two terrestrial recording locations (Supplementary Fig. S1–S2).

## Discussion

Investigation of the variations of biological sounds is an essential task in evaluating the behaviour of calling animals on the basis of acoustic recordings^13^. In this study, we demonstrated that the PC-NMF is useful for separating a biological chorus from a long-term spectrogram containing multiple sound sources, including environmental and anthropogenic noises. The PC-NMF acts as an automatic denoising algorithm, which improves the investigation of the occurrence of calling animals and the structural change of animal vocalizations in long-duration recordings.

Although our simulation analysis indicated that the performance of PC-NMF was not as robust as that of the supervised NMF, the PC-NMF still provided a solution to enhance biological choruses without any training data. The preparation of training data is an important issue in the long-term and large-scale acoustic monitoring of biodiversity, because it is very difficult to collect sufficient training data with good recording quality. The other unsupervised spectral restoration approaches, such as the MLSA, MAPA, and GMAPA spectral restoration methods, estimate the gain function in the spectral domain on the basis of probabilistic distribution models of the acoustic signal and noise^20–23^. However, the spectral restoration methods cannot identify different types of noise in long-term field recordings. Although they are efficient in decreasing stationary noise, they are unable to handle the transient noise produced by weather events and human activities.

There are two primary advantages of the PC-NMF proposed in this study. The first advantage is the self-learning ability of PC-NMF, which allows it to decompose a spectrogram into a basis matrix and encoding matrix. The encoding matrix provides preliminary information regarding the temporal change of each sound source so that the second NMF can be used to group different bases. The second advantage is that PC-NMF can provide unsupervised clustering of the basis matrix according to the periodicity measured from the encoding matrix. Because of the complexity of animal vocalizations, different biological sounds may display varied spectral characteristics, but most of the biological sounds occur with a periodical pattern. Therefore, the periodicity measured from the encoding matrix can aid in differentiating whether a basis belongs to the biological chorus with evident diurnal behaviour or the other noise source without a similar occurrence pattern.

The PC-NMF has been tested in both quiet and noisy environments, and its performance relies on the periodicity-based basis clustering. However, all the unsupervised clustering algorithms possess uncertainty, owing to the lack of training data. Our observations also suggest that the PC-NMF may not perform well when the periodicity of biological sounds in the testing data is not very prominent. The calling of many animals has a seasonal pattern, e.g., cicada calls may be recorded only during warm seasons. Therefore, precautions must be taken in processing a year-long dataset. The periodicity may be decreased when a biological sound is not recorded over the entire year. After the periodicity is reduced, the unsupervised clustering of the basis matrix may not be accurate, thus lowering the NR capability of PC-NMF. Therefore, it is recommended to first use PC-NMF for a short spectrogram fragment with a prominent biological chorus to confidently obtain the basis matrix for biological and non-biological signals. The encoding matrix of the entire dataset can be subsequently analysed by using the combined basis matrix collected from different spectrogram fragments. Finally, the biological chorus in a long-term dataset can be reconstructed by using the supervised NMF approach.

In addition to enhancing the biological chorus and suppressing noise sources, PC-NMF can also be revised so that it can recognize individual sound sources. To achieve this, more features, such as the spectral characteristic and spectral modulation, should be used in the unsupervised basis clustering. Considering the simulated long-term spectrogram (Fig. 1) as an example, there were five sound sources with unique spectral and temporal characteristics. During our preliminary analysis, we combined information collected from the encoding matrix, modulation of the encoding matrix on the basis of discrete Fourier transform, basis matrix, and modulation of the basis matrix during the second stage of PC-NMF to classify the basis matrix into five basis clusters. The results indicated that the five sound sources could be effectively separated by PC-NMF in an unsupervised manner (please refer to Supplementary Fig. S3). MATLAB codes of the PC-NMF proposed in this study are provided in Supplementary Dataset S1. We encourage researchers to test the performance of PC-NMF using real-world data and to develop other extensions of PC-NMF to facilitate the acoustic-based biodiversity monitoring in the future.

## Conclusions

With the proposed PC-NMF algorithm, biological choruses can be effectively separated from a noisy long-term spectrogram without any training data. The source separation technique can then be used as a denoising algorithm to enhance the biological choruses. A simple index, such as the intensity and complexity of acoustic data, may describe the dynamics of calling animals with less bias and without the need to expend explicit effort to design detectors and classifiers. Therefore, the passive acoustic monitoring of calling animals is possible even in noisy environments, such as a remote island with strong environmental noise or an urban park with noticeable anthropogenic noise. In addition to the enhancement of biological choruses, the environmental noise and anthropogenic noise separated by the PC-NMF may also be considered as indicators of habitat quality and human activities. In the future, the interactions among habitat, animal behaviour, and human disturbance can be investigated through long-term acoustic monitoring and the separation of environmental noise, biological sounds, and anthropogenic noise.

## Methods

In this study, long-term spectrograms from long-duration recordings were used as the input data to separate different sound sources and suppress noise components from the signals of interest. The use of a spectrogram is an efficient way to visualize acoustic data in the frequency and time domains. However, it is impractical to visualize a large amount of acoustic data by using a spectrogram based on the short-time Fourier transform. Instead, the mean or median power spectrum can be measured as a representation of each recording file, and the variation across multiple recording files can then be investigated^30^. In this study, we primarily used a median power spectrum to demonstrate the application of PC-NMF. With a long-term spectrogram, a biological chorus, which occurs periodically, can be easily observed^31^. Below, we describe the methodology of using PC-NMF to separate biological sounds with strong periodicity from a long-term spectrogram.

### Non-negative matrix factorization

NMF is a self-learning algorithm that learns parts-based representation from a non-negative matrix^27, 28^. Although a spectrogram may be negative if the acoustic data are not calibrated on the basis of the sensitivity of the microphone or hydrophone used during the spectral analysis, it can still be transformed into a non-negative matrix via a simple linear transformation. Through iteration of the update procedures, NMF determines an approximate factorization of the non-negative matrix (*V*), on the basis of the other two non-negative matrices, *W* and *H* ^27^.

1 $${V}_{ij}\approx {(WH)}_{ij}=\sum _{a=1}^{r}{W}_{ia}{H}_{aj}$$

where *V*_*ij*_ is the *ij*-th component of *V* (matrix that contains multiple sound sources) and *W*_*ia*_ and *H*_*aj*_ are the *ia*-th component of *W* (the basis matrix) and the *ai*-th component of *H* (the encoding matrix), respectively. The basis matrix can be considered the building block for reconstructing *V*, and the encoding matrix describes the strength of each building block. Through NMF, each vector in *V* can be modelled by *W* and the corresponding *H*.

The constraint of a non-negative matrix assumes that the data are a combination of multiple sources, thus reflecting the additive nature of sounds in nature environments. This constraint makes NMF an effective method for finding a meaningful approximation of the basis matrix for different sound sources^27^. Each basis often corresponds to the spectral features of a sound source or at least part of a sound source. In contrast, *H* can be used to indicate the relationship between each data vector and each basis if a sparseness constraint is incorporated during the matrix factorization^32^. Therefore, the NMF has also been used in the automatic transcription of polyphonic music or event clustering^33, 34^.

### Non-negative matrix factorization-based noise reduction

**Figure 7:**
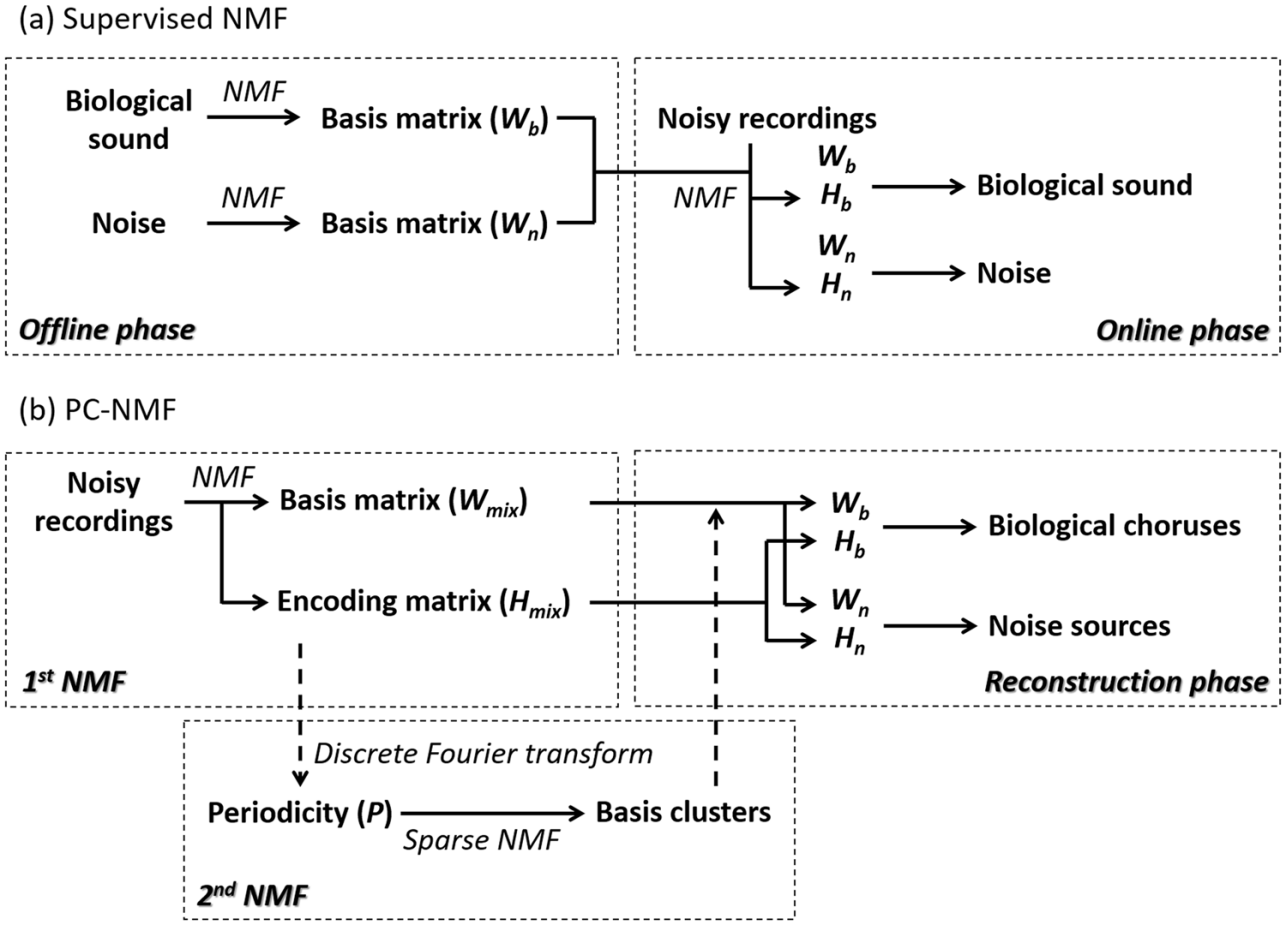
Analysis procedures of NMF-based noise reduction. (**a**) Supervised NMF is composed of an offline phase and online phase. (**b**) PC-NMF is composed of two stages of NMF and one reconstruction stage.

The noise reduction of supervised NMF consists of two phases: offline and online. In the offline phase, users must prepare training data for different sound sources to obtain their basis matrices. In the example of Fig. 7a, two basis matrices for biological sound (*W*_*bio*_) and noise (*W*_*noise*_) are modelled individually. After the basis matrices are obtained for the biological sound and noise, a new encoding matrix (*H*_*mix*_) can be trained for a noisy recording, on the basis of the combined basis matrix (*W*_*mix*_) in the online phase. Then, the biological sound can be separated from the noise by multiplying *W*_*bio*_ and the associated *H*_*bio*_.

In PC-NMF, there are no training data available for preparing the basis matrices. A basis matrix *W*_*mix*_ and an encoding matrix *H*_*mix*_ are directly obtained from a noisy recording. The critical function of PC-NMF is to precisely separate *W*_*bio*_ and *W*_*noise*_ from *W*_*mix*_ without any prior knowledge of the two sound sources. Figure 7b shows the procedures of the PC-NMF proposed in this study, which include three stages: the first stage decomposes the input spectrogram into a basis matrix (*W*_*mix*_) and an encoding matrix (*H*_*mix*_). The second stage extracts a periodicity matrix *P* by applying periodicity analysis to *H*_*mix*_:

2 $$P={F}_{PA}({H}_{mix})$$

where *P* is the periodicity matrix, which carries the periodicity information of the mixed data (as shown in Fig. 2d), and *F*_*PA*_(·) denotes the periodicity analysis function. In this study, we used the discrete Fourier transform.

On the basis of the periodicity matrix, the bases in *H*_*mix*_ can be separated without supervision into two clusters by using the sparse NMF clustering method^32, 34^ as follows

3 $${H}_{p}=\mathop{{\rm{argmin}}}\limits_{{H}_{p} > 0}[d({P}^{T},{W}_{p}{H}_{p})+\lambda ||{H}_{p}|{|}_{1}]$$

where *λ* represents the sparsity penalty factor, ||·||_1_ represents the L1-norm, and *d*(·) is a distance measure. On the basis of the largest score within the encoding matrix, *H*_*p*_, of the transposed periodicity matrix, *P* ^*T*^, the clustering assignment of *H*_*mix*_ can be determined. Finally, *W*_*mix*_ is separated into biological and non-biological basis clusters according to the clustering assignment of *H*_*mix*_. The basis cluster with stronger periodicity is considered the essential component to reconstruct the biological chorus from field recordings. However, the basis cluster with weaker periodicity is considered to be associated with environmental or anthropogenic noise. In the third stage, the separated *W*_*bio*_ and *W*_*noise*_ with their corresponding encoding components in *H*_*mix*_ are then used to reconstruct the biological choruses and noise signals.

### Performance evaluation of noise reduction

The performance of PC-NMF was evaluated by using one simulated long-term spectrogram and four real-world datasets. The simulated long-term spectrogram was composed of five sources, including three biological sounds, one low-frequency noise, and one broadband noise (Fig. 1). The three biological sounds occupied different frequency ranges: low-frequency, middle-frequency, and high-frequency. All three types of biological sound occurred with a strong periodical pattern but appeared in different time periods to simulate the diurnal behaviour of various calling animals. The low-frequency noise was simulated as the environmental noise, which occurred in a stationary pattern. Finally, the broadband noise was simulated as a transient noise event, which masked the biological sounds recorded from the low- to middle-frequency ranges.

By using the simulated long-term spectrogram, we compared the separation performance between the supervised NMF, PC-NMF, and conventional spectral restoration methods. The evaluation was measured on the basis of the spectral similarity, *r*, the mean of correlations between each vector of the original spectrogram (*O*) and separated spectrogram (*V*):

4 $$r=\frac{{\sum }_{j=1}^{n}Corr({O}_{j},{V}_{j})}{n}$$

where *Corr*(·) denotes the correlation function and *O*_*j*_ and *V*_*j*_ are the *j*-th components of *O* and *V*, respectively. The spectral similarity is a value between −1 and 1; higher similarity suggests that the separated data have a better correlation with the ground truth. In contrast, a poor separation results in a spectral similarity lower than or close to 0.

### Application of PC-NMF to real-world data

In addition to the simulated spectrogram, long-duration recordings collected in different marine and terrestrial environments were also used in this study to test the performance of PC-NMF. Underwater recordings were acquired with SM2M submersible recorders (Wildlife Acoustics Inc., Maynard, MA) at two locations. The first recording location was of the shallow waters near the Waisanding Sandbar in western Taiwan, where the water depth ranged from 2 to 5 m. The Waisanding Sandbar is a relatively pristine environment with a small number of fishing boats that pass by each day. The other recording location was the Xinhuwei River Estuary, also in western Taiwan, where the water depth ranged from 8 to 12 m. This recording location was close to an industrial harbour, and the underwater environment was relatively noisy, owing to the operation of several petrochemical factories and frequent cargo shipping.

Terrestrial recordings were made by using SM2+ and SM4 acoustic recorders (Wildlife Acoustics Inc., Maynard, MA) at two locations. The first recording location was Lienhuachih, a subtropical, evergreen, broad-leaved forest located in Nantou County in central Taiwan. The altitude of the recording location was approximately 800 m. Lienhuachih is a research centre of forestry ecology. Only a few researchers at a time visit the recording location. The second recording location was the Guandu Nature Park, a protected wetland located in the northwestern part of Taipei City, which is the largest city in Taiwan. Although the Guandu Nature Park is a protected wetland, there is one main road passing through its northern boundary. In addition, there is also a gravel plant nearby; thus, the background sound at the Guandu Nature Park is mostly dominated by anthropogenic noise.

## Electronic supplementary material

Supplementary information

Dataset 1
