# Supplementary material for: Improving biodiversity assessment via unsupervised separation of biological sounds from long-duration recordings
Source: Sci Rep. 2017 Jul 3;7:4547. doi: 10.1038/s41598-017-04790-7 (PMC5495775; doi:10.1038/s41598-017-04790-7)
Supplement: Supplementary file 1 — Supplementary information [file 41598_2017_4790_MOESM1_ESM.pdf]

Improving biodiversity assessment via unsupervised separation of biological sounds from long-duration recordings

AUTHOR INFORMATION

Tzu-Hao Lin[1*], Shih-Hua Fang[2], Yu Tsao[1*]

[1]Research Center for Information Technology Innovation, Academia Sinica

[2]Department of Electrical Engineering, Yuan Ze University

CONTACT INFORMATION

*corresponding e-mail addresses: schonkopf@gmail.com (THL), yu.tsao@citi.sinica.edu.tw (YT)

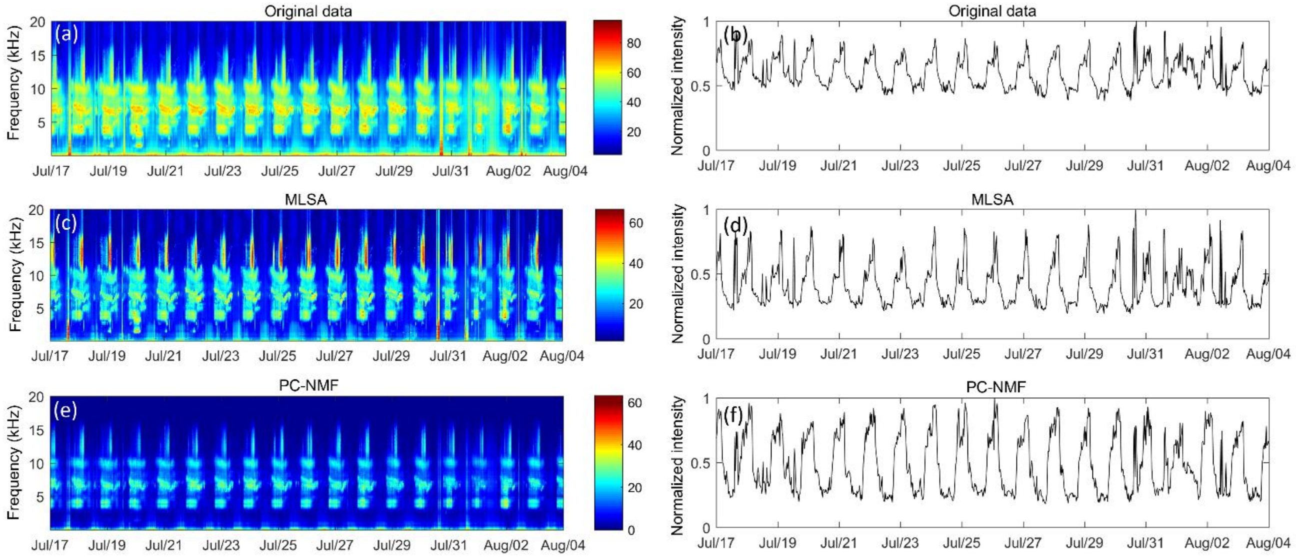

Supplementary Figure S1. Spectrograms and normalized intensities of terrestrial recordings collected at the Guandu Nature Park. The graph shows a comparison between (a-b) the original data and the analysis results using (c-d) the MLSA method and (e-f) the PC-NMF.

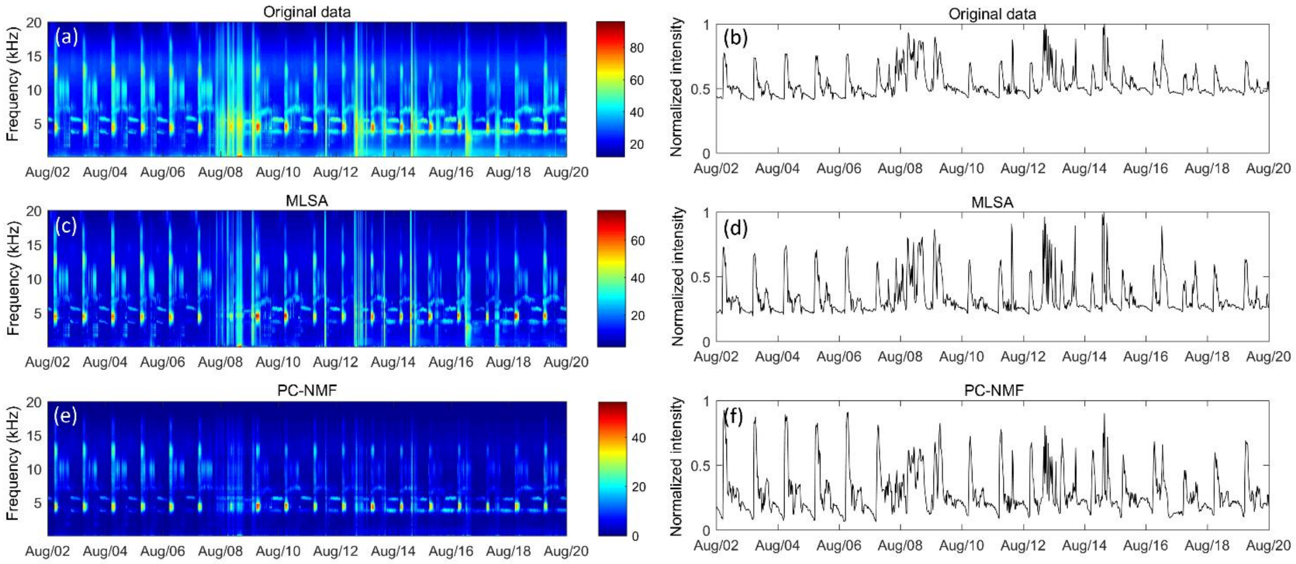

Supplementary Figure S2. Spectrograms and normalized intensities of terrestrial recordings collected at the Lienhuachih. The graph shows a comparison between (a-b) the original data and the analysis results using (c-d) the MLSA method and (e-f) the PC-NMF.

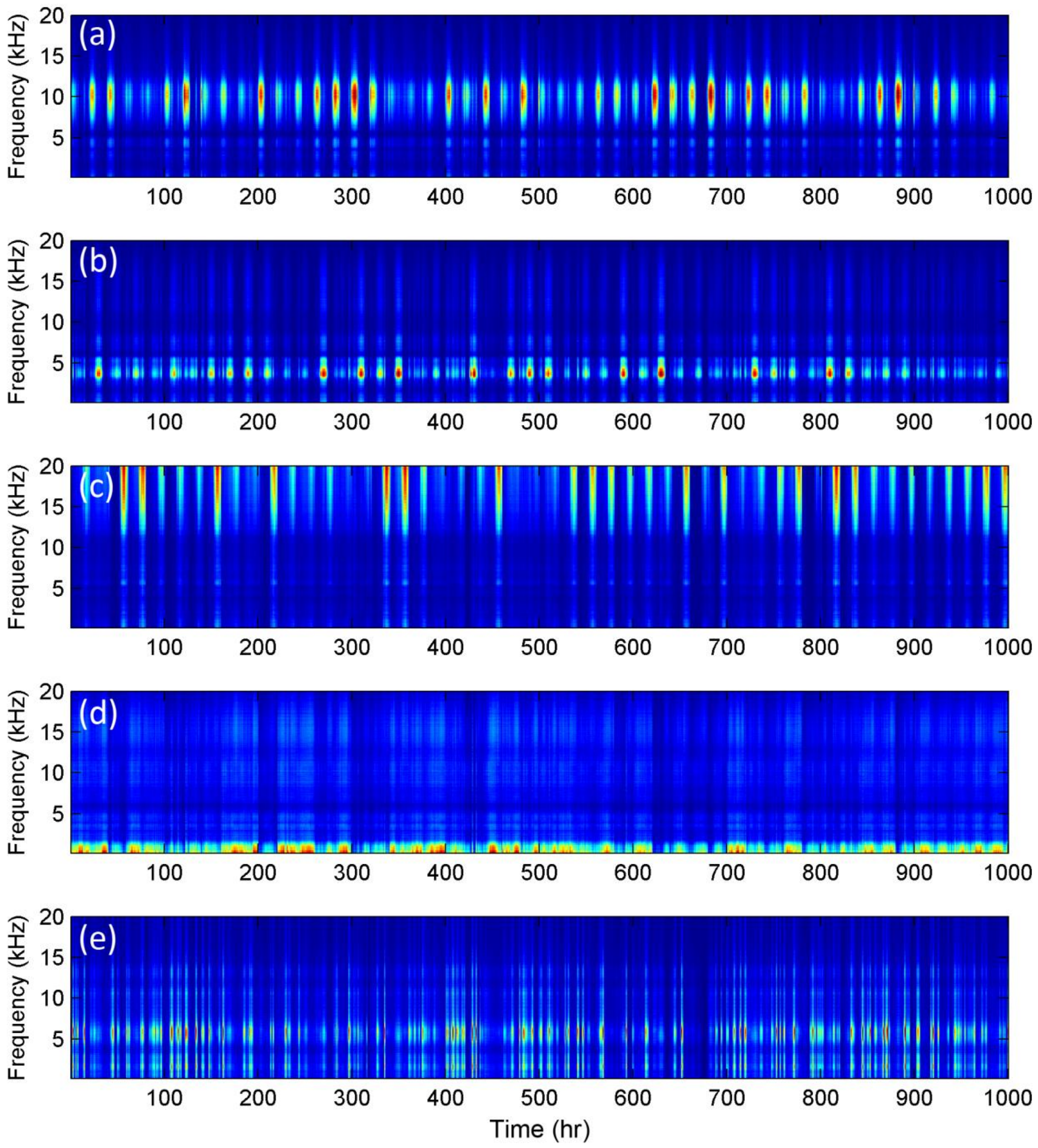

Supplementary Figure S3. Spectrograms of the five sound sources separated by using the PC-NMF. The result was produced by using the same PC-NMF algorithm, however, we expand the input features (encoding matrix, modulation of encoding matrix based on discrete Fourier transform, basis matrix, and modulation of basis matrix during the second stage of PC-NMF to recognize five individual sources.

Supplementary File S1. MATLAB codes of separating different sound sources using the PC-NMF. Includes the primary MATLAB function to run PC-NMF (LTSA_PCNMF.m), all long-term spectrograms tested in this study (data.mat) and code examples of using PC-NMF to process different long-term spectrograms (PCNMF_test.m).
